# P-1255. A Vancomycin Pharmacokinetic Model for Adults with Low Serum Creatinine Derived from a Large Multisite Database

**DOI:** 10.1093/ofid/ofaf695.1446

**Published:** 2026-01-11

**Authors:** Maria-Stephanie Hughes, Jon Faldasz, Jordan Brooks

**Affiliations:** InsightRX, Boston, MA; InsightRX, Boston, MA; InsightRX, Boston, MA

## Abstract

**Background:**

Low muscle mass is associated with low serum creatinine (SCr), leading to overestimated renal function and reduced accuracy in vancomycin pharmacokinetic (PK) models. Pharmacists often round up low SCr, which can introduce bias. We developed and evaluated a model trained on patients with low SCr.

**Table 1.**
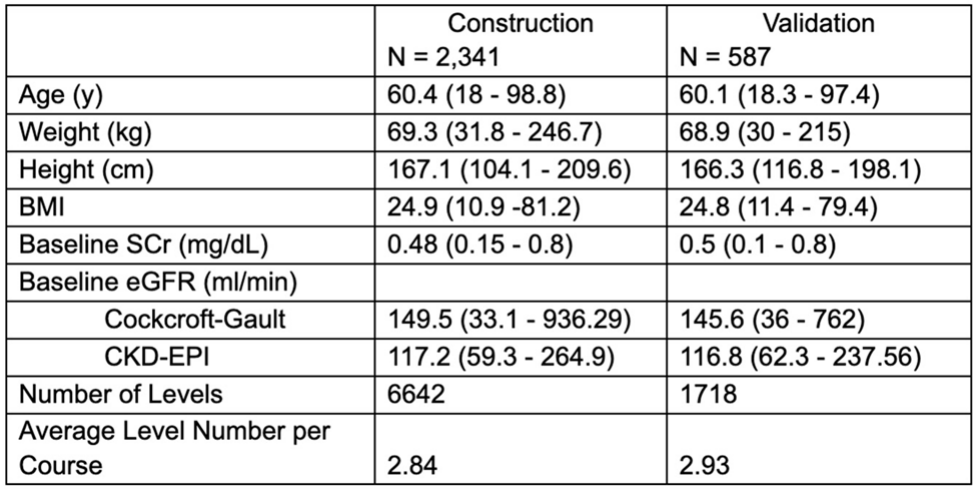
Patient Characteristics Patient characteristics are shown for the model construction dataset as well as the validation dataset. All reported values are the median (range) unless otherwise specified as a number count.

**Table 2.**
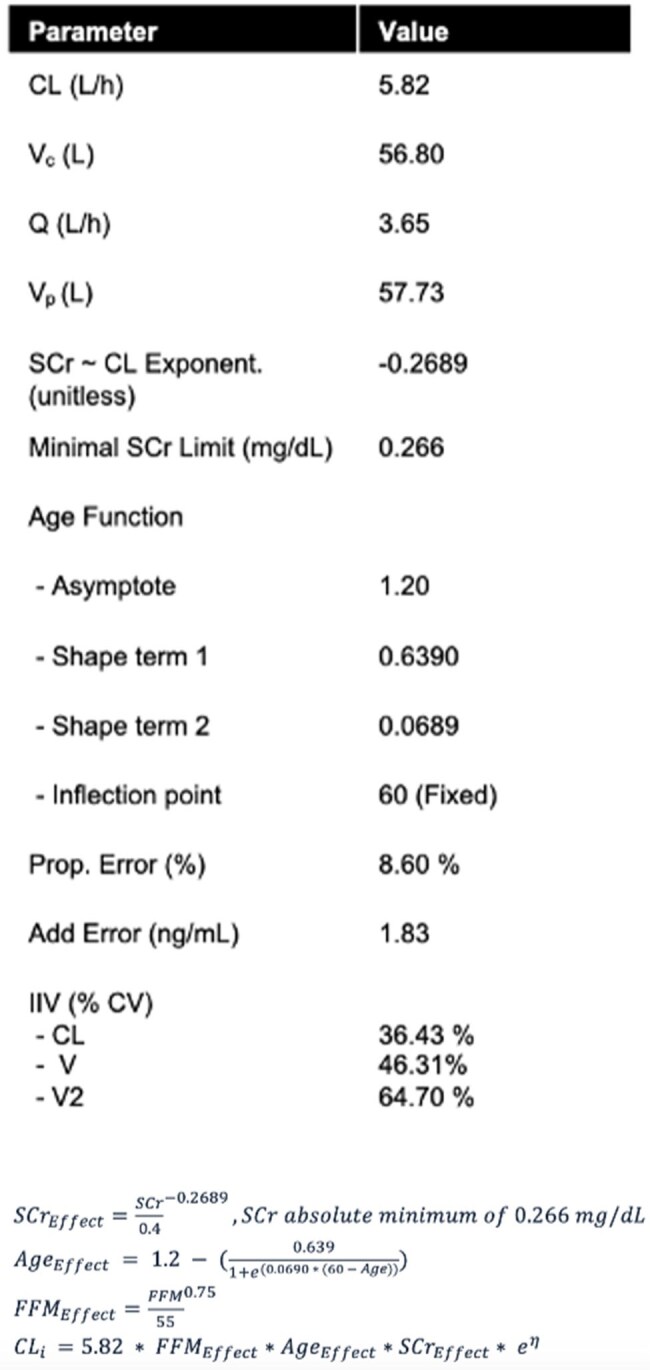
Final Parameter Estimates and CLvanco Equations The final model’s pharmacokinetic parameter estimates are shown in the table, and the final vancomycin clearance (CLvanco) equations are shown at the bottom of the table. Abbreviations: CL= Population clearance; Vc and V= volume of distribution of the central compartment; Q= rate of flow between the central and peripheral compartments; Vp and V2= volume of distribution of the peripheral compartment; Prop.= proportional; IIV= inter-observation variability; CV= coefficient of variation; FFM= fat free mass; CLi= Individual Clearance.

**Methods:**

Data from adults >18 years receiving vancomycin from Jan 2021–Jan 2025 were retrospectively collected from a multisite Bayesian database. Inclusion required SCr < 0.8 mg/dL, ≥1 vancomycin level, ≥3 doses, and ≥2 days of therapy. Exclusions included pregnancy, renal replacement therapy, extracorporeal membrane oxygenation, amputations, and para/quadriplegia. Patients were matched and split into training and test datasets. Those with SCr 0.6–0.8 mg/dL were down-sampled to prioritize those with lower SCr. Modeling and forecasting were performed using NONMEM v7.6 (FOCE-I) and PsN proseval. Covariate testing included CrCl/eGFR equations (e.g., Cockcroft-Gault, CKD-EPI), raw SCr, body size indicators (e.g. total body weight, fat free mass), and age. Performance of the final model was compared to 6 published models by measuring bias with mean percent error (MPE), imprecision with normalized root-mean square error (nRMSE), and accuracy (within 15% or 2.5 mg/dL), both *a priori* and *a posteriori*. Predictive comparisons were also made using adjusted SCr values (AdjSCr = [Measured SCr/2] + 0.5) and rounded values (1.0, 0.8, 0.6, or 0.4 mg/dL) vs. measured SCr values.

**Figure 1.**
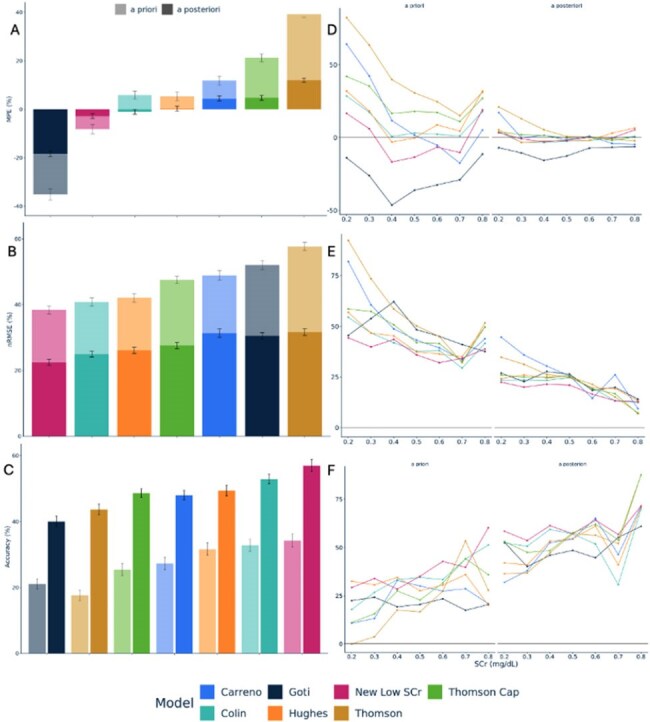
Model Performance Model performance is forecasted as grouped by models (labeled by first author’s last name of publication and with colors) and prediction type (a priori vs a posteriori) (A-C) or as further grouped rounded SCr value (D-F). Evaluated in terms of bias with MPE (A,D) ,imprecision with nRMSE (B,E), and Accuracy (C,F). Thomson Cap refers to a modified version of the model by Thomson et al. that includes a capped eGFR of 150 ml/min.

**Figure 2.**
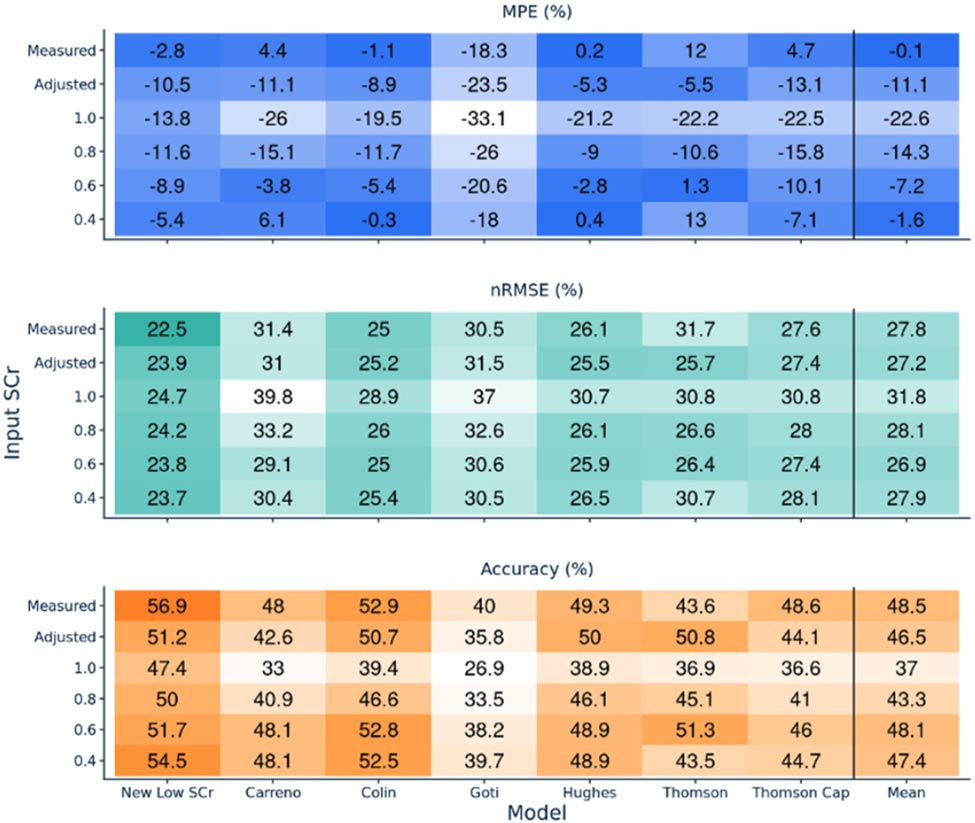
Model Performance Based on Input SCr Model performance is forecasted as grouped by model (labeled by the first author’s last name of publication) and input SCr (as measured, adjusted, or rounded to specified values) with reported bias with MPE, precision with nRMSE, and Accuracy and darkness of shading corresponding to better performance in each metric, respectively. Thomson Cap refers to a modified version of the model by Thomson et al. that includes a capped eGFR of 150 ml/min.

**Results:**

The construction dataset included 2,341 patients (6,642 levels), and validation included 587 patients (1,718 levels); median SCr was 0.5 mg/dL (range: 0.1–0.8). The final model retained SCr and age as covariates, without incorporation into CrCl/eGFR equations, and set a minimum SCr of 0.266 mg/dL. It showed the best overall performance, though two models (Colin et al. and Hughes et al.) performed similarly. Measured SCr yielded the best results for the developed model but not consistently for others.

**Conclusion:**

The developed low SCr vancomycin PK model outperformed 6 published models and holds promise in significantly improving PK predictions and dosing decisions when utilizing it in a Bayesian dosing software in this population where dosing is typically challenging.

**Disclosures:**

Maria-Stephanie Hughes, PharmD, InsightRX: Employee of company|InsightRX: Stocks/Bonds (Private Company) Jon Faldasz, PharmD, BCPS, InsightRX: Employee Jordan Brooks, PharmD, InsightRX: Employee of company|InsightRX: Stocks/Bonds (Private Company)

